# H_2_S- and Redox-State-Mediated PTP1B S-Sulfhydration in Insulin Signaling

**DOI:** 10.3390/ijms24032898

**Published:** 2023-02-02

**Authors:** Yu-Chin Lin, Wan-Ting Zeng, Der-Yen Lee

**Affiliations:** 1 Ph.D. Program for Health Science and Industry, China Medical University, No. 91, Hsueh-Shih Road, Taichung 40402, Taiwan; 2 Graduate Institute of Integrated Medicine, China Medical University, No. 91, Hsueh-Shih Road, Taichung 40402, Taiwan

**Keywords:** H_2_S, S-sulfhydration, PEG-switch, PTP1B, insulin, glutathione, redox

## Abstract

Because hydrogen sulfide (H_2_S) is classified as a gaseous signaling molecule, protein S-sulfhydration is known to be one of the mechanisms by which H_2_S signals are conducted. PTP1B, a negative regulator in insulin signaling, has been found to be S-sulfhydrated at Cys215-SH to form Cys215-SSH in response to endoplasmic reticulum (ER) stress. Therefore, we aimed to understand the change in PTP1B S-sulfhydration and cellular redox homeostasis in response to insulin stimulation. We demonstrated a feasible PEG-switch method to determine the levels of PTP1B S-sulfhydration. According to the results obtained from HEK293T and MDA-MB-231 cells, insulin induced a change in PTP1B S-sulfhydration that was similar to the change in Insulin receptor substrate 1 (IRS1) phosphorylation in both cell lines. However, insulin-induced PTP1B S-sulfhydration and IRS1 phosphorylation were only significantly affected by metformin in HEK293T cells. Insulin also induced an increase in reactive oxygen species (ROS) in both cell lines. However, the level of H_2_S, GSH, and GSSG was only significantly affected by insulin and metformin in HEK293T cells. HEK293T cells maintained high levels of H_2_S and cysteine, but low levels of GSSG and GSH in general compared to MDA-MB-231 cells. From these findings, we suggest that PTP1B activity is modulated by H_2_S and redox-regulated S-sulfhydration during insulin signaling.

## 1. Introduction

Hydrogen sulfide (H_2_S) is categorized as one of the gaseous messenger molecules, which include nitric oxide and carbon monoxide. Cellular H_2_S is primarily generated from the metabolism of cysteine, homocysteine, and cystathionine, catalyzed by cystathionine β-synthase (CBS), cystathionine γ-lyase (CSE), or 3-mercaptopyruvate sulfurtransferase (MST), and cysteine is the pivot precursor for producing H_2_S [1,2,3,4,5]. Various cytoprotective functions of H_2_S, such as antioxidant defense, vasorelaxation, neurotransmission, thermotolerance, and increasing insulin receptor sensitivity, have previously been described [6,7]. One of the mechanisms by which H_2_S signals are conducted is mediated by protein S-sulfhydration of cysteine residues, which usually possess a low p*K*a value [8]. Protein cysteine residues with low p*K*a are prone to dissociating into thiolate (P-S^−^) at physiological pH. Thiolate is reactive to reactive oxygen species (ROS) such as H_2_O_2_ to form a protein-sulfenic acid (P-SOH) or be irreversibly modified to higher oxidation states, such as into a protein-sulfinic acid (P-SO_2_H) or a protein-sulfonic acid (P-SO_3_H) [9,10,11]. A protein-sulfenic acid (P-SOH) can be further converted to a protein-persulfide (P-SSH) through reaction with HS^−^ or H_2_S [12].

Protein S-sulfhydration mediated by H_2_S participates in cellular regulation by changing the functions of proteins, such as GAPDH (glyceraldehyde-3-phosphate dehydrogenase), nuclear factor-κB (NF-κB), and protein tyrosine phosphatase 1B (PTP1B) [1,12,13,14,15,16,17]. PTP1B contains an active site at the cysteine residue (Cys215) that removes the phosphate from the substrate phosphotyrosyl residues. Since PTP1B activation stops insulin and leptin signaling, maintaining the inactivation of PTP1B may be applied therapeutically to improve signaling sensitivity. Compounds such as thiazolidinediones (TZDs), phosphorus-containing phosphotyrosyl mimetics, and trodusquemine (MSI-1436), which possess inhibitory characteristics on PTP1B, have been screened and used to treat type 2 diabetes [18,19,20,21]. Anti-oxPTP1B antibody has also been developed and demonstrated to enhance insulin signaling in HEK293T cells in a redox-dependent manner [22,23]. The function of PTP1B is regulated by NADP oxidase (NOX) oxidation and cellular reduction. All cysteine modifications are recoverable except for the biologically irreversible modifications into protein-sulfinic acid and protein-sulfonic acid. The thioredoxin system, accompanied by glutathione and reductive coenzymes, plays an essential role in regulating PTP1B activity in signaling, e.g., the thioredoxin system selectively activates oxidized PTP1B to modulate PDGF-β receptor tyrosine kinase signaling [24]. Moreover, in response to endoplasmic reticulum (ER) stress, H_2_S is capable of creating PTP1B S-sulfhydration at Cys215-SH to form Cys215-SSH, further inactivate activity, and maintain PERK in its active and phosphorylated form [12].

To date, several methods based on chemical modifications have been developed to monitor protein S-sulfhydration [25]. Firstly, a method modified from the biotin switch assay permits the detection of protein S-sulfhydration [14]. Protein S-sulfhydration can also be identified by applying maleimide, which specifically reacts to sulfhydryl groups of cysteine residues without affecting nitrosylated or oxidized cysteine residues. An alternative method proposed by Sen et al. (2012) for persulfide detection is based on the fact that both persulfide and free thiol are blocked by the thiol-blocking reagent N-ethyl maleimide [17]. In brief, Cy5-conjugated maleimide is used in the first step, followed by the use of dithiothreitol (DTT) in the second. Protein persulfides react to Cy5-maleimide to form a disulfide, and are cleaved by DTT, leading to a decrease in the in-gel fluorescence signal, but not the sulfide formed by protein thiols and Cy5-maleimide. This method is relatively simple, and the reagents are commercially available [17,25]. In 2019, Lee and Chang proposed a method for quantitatively displaying the redox status of proteins with maleimide-polyethylene glycol (mPEG) tagging, and surveyed the redox state of proteins in H_2_O_2_-, epidermal growth factor (EGF)-, and insulin-treated cells [26]. This method is also simple, and the reagents are also commercially available. More importantly, the PEG-switch method is applicable for the detection of S-sulfhydrated proteins. Theoretically, the PEG-switch method can be used to determine S-sulfhydration for any protein of interest by the procedure of mPEG labeling and SDS-PAGE or immunoblotting.

The widely prescribed insulin sensitizer metformin exerts pleiotropic actions on multiple tissues by partially recognized mechanisms. One of the actions found for metformin is to increase H_2_S tissue concentrations in mouse brain, heart, kidney and liver tissues [27]. The reduced production of H_2_S through the administration of methionine can be reversed by metformin by regulating CSE expression [28]. Metformin is used to improve insulin sensitivity in insulin-resistant (IR) conditions such as diabetes, prediabetes, polycystic ovary syndrome, and obesity. Although the correlation between glucose transporter 4 (GLUT4) expression and the level of IR has been described, the mechanisms of metformin behind this modulation are still largely undefined [29]. On the other hand, despite studies having indicated that metformin may be a promising chemotherapeutic agent, the effects and underlying mechanisms of metformin on anti-neoplasia have not been fully elucidated [30]. To uncover the undefined effects of metformin on insulin signaling and the difference between cancer cells and non-cancer cells, we selected HEK293T, a human embryonic kidney cell line, and MDA-MB-231, a human breast cancer cell line, to examine the effects of PTP1B S-sulfhydration, redox state, and thiol metabolite contents under insulin and metformin treatment.

In this article, we applied the PEG-switch method with non-reducing and reducing SDS-PAGE to resolve the PTP1B S-sulfhydration, and further revealed the regulation of PTP1B activity under insulin stimulation. It was found that the levels of S-sulfhydrated PTP1B in HEK293T or MDA-MB-231 cells varied with the progression of time with insulin stimulation, both in the presence and absence of metformin. HEK293T cells exhibited a high capacity to generate ROS and H_2_S, which prevalently resulted in a significant inhibition of PTP1B activity by S-sulfhydration. The contents of H_2_S and cysteine in HEK293T cells were much higher than those in MDA-MB-231 cells. Conversely, the contents of GSSG and GSH in HEK293T cells were much lower than those in MDA-MB-231 cells. Moreover, the level of H_2_S in HEK293T cells was susceptible to being affected by treatment with insulin and metformin, compared to that in the MDA-MB-231 cells. Therefore, the thiol metabolites may also be relevant in maintaining the homeostasis of PTP1B S-sulfhydration. In general, we demonstrated a feasible method for determining protein S-sulfhydration and revealed that PTP1B S-sulfhydration mediated by H_2_S and redox state was involved in the response and regulation in insulin signaling.

## 2. Results

### 2.1. Using the PEG-Switch Method to Detect Protein S-Sulfhydration

**Figure 1:**
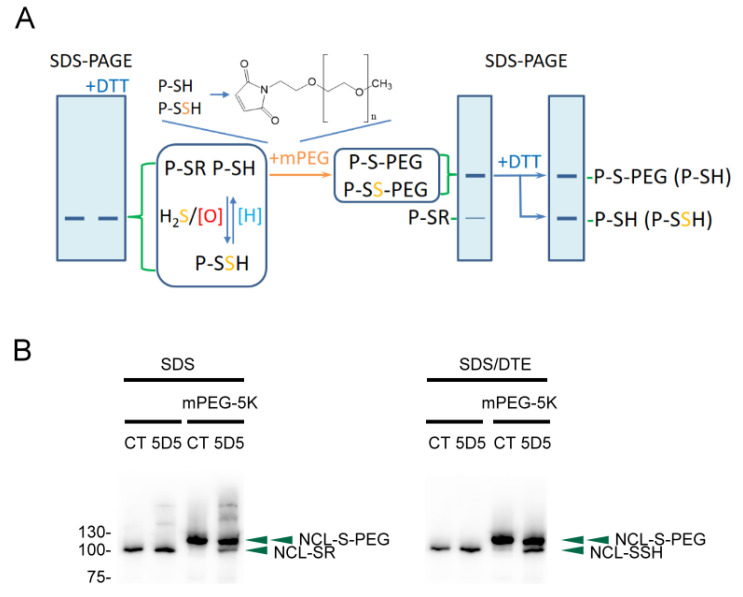
Tag-shift technique for determining protein S-sulfhydration. (**A**) The procedure of labeling and detection. (**B**) Nucleolin (NCL) S-thiolation and S-sulfhydration in diamide-treated FaDu cells. P-SR: S-thiolated proteins; P-SH: proteins with a thiol; P-SSH: S-sulfhydrated proteins; [O]: oxidants; [H]: reductants; mPEG: methoxypolyethylene glycol maleimide (5 kDa); DTT: dithiothreitol.

Protein thiols (P-SH) can be oxidized to protein sulfenic acids (P-SOH) by H_2_O_2_ or ROS, which can react with H_2_S to form protein persulfides (P-SSH). Both protein persulfides and thiols are reactive to mPEG by nucleophilic addition, and the other S-thiolated proteins remain unreactive. The mPEG-tagged proteins would be significantly retarded in electrophoretic migration due to the additional 5 kDa of molecular weight with each mPEG labeling, and the other S-thiolated proteins would remain at the original position. For mPEG-labeled P-SH and P-SSH, only the disulfide bonds on P-SS-PEG can be reduced with reducing agents, e.g., DTT, to form a reduced protein (P-SH). Therefore, the protein bands representing P-SSH would drop to a lower molecular weight in reducing SDS-PAGE compared to those representing P-SH, and the relative protein persulfides in each sample can be determined using this PEG-switch method (Figure 1A). We applied the method to detect the S-sulfhydrated proteins in the FaDu cells treated with diamide (tetramethylazodicarboxamide, TMAD), an oxidizing agent. Among the PEG tagged proteins, nucleolin has only one cysteine residue at Cys543, and the S-thiolated and S-sulfhydrated nucleolin was determined by using this method with immunoblotting (Figure 1B). Thus, the method is feasible for selectively identifying the S-sulfhydrated protein from a protein pool.

### 2.2. The S-Sulfhydration and Activity of Na_2_S-Treated Recombinant PTP1B

**Figure 2:**
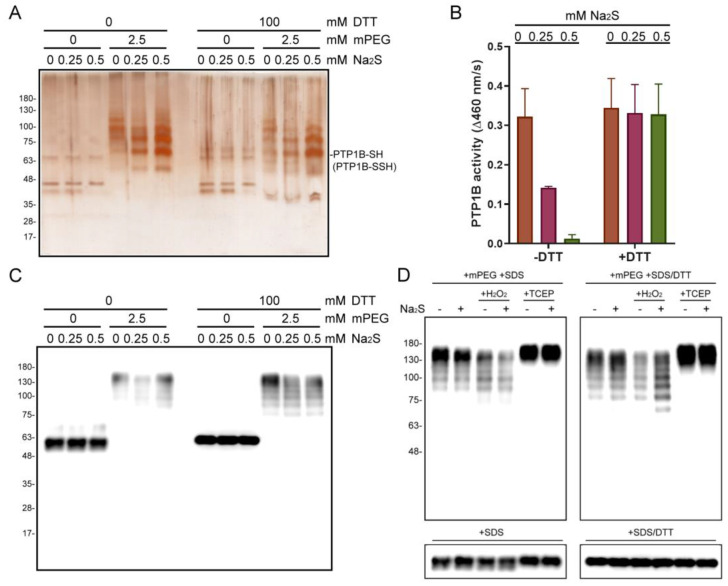
In vitro PTP1B S-sulfhydration. (**A**) Detection of S-sulfhydrated PTP1B in Na_2_S-treated recombinant PTP1B samples by tag-shift technique. (**B**) The related PTP1B activity of Na_2_S-treated protein samples. (*n* = 3). (**C**) Detection of S-sulfhydrated PTP1B in Na_2_S treated HEK293T cell lysates. (**D**) Detection of S-sulfhydrated PTP1B in Na_2_S-treated HEK293T cell lysates with pre-treatment of H_2_O_2_ or TCEP.

To survey the properties of S-sulfhydration of PTP1B, we firstly performed an in vitro reaction by incubating recombinant human PTP1B with Na_2_S and then subjecting it to mPEG labeling. The protein samples were then subjected to SDS-PAGE with silver staining. In the part of gel without the presence of DTT in samples, the PEG-labeled PTP1B was differentially retarded in the gel, and no PTP1B band was detected at the original position of molecular weight. With the presence of DTT, the reduced PTP1B band was then detected at the position near the unlabeled PTP1B, and the quantity of reduced PTP1B was proportional to the concentration of Na_2_S (Figure 2A). The duplicate samples were also subjected to PTP activity assays. The data showed that the presence of Na_2_S suppressed the PTP1B activity, which was recovered by administrating DTT (Figure 2B). HEK293T cell lysate was also used to carry out in vitro sulfhydration, and a similar result was acquired with the immunoblot of PTP1B (Figure 2C). In addition, the H_2_O_2_ and TCEP pre-treated cell lysates were also applied to monitor the effect of redox state on PTP1B S-sulfhydration. As a result, the presence of H_2_O_2_ promoted the PTP1B S-sulfhydration with Na_2_S; however, the addition of TCEP resulted in the retention of all PTP1B as PEG-tagged adducts (Figure 2D). These results indicate that H_2_S and oxidative conditions are required to catalyze the S-sulfhydration of PTP1B, and the inhibited enzyme activity is able to be recovered by reducing agents.

### 2.3. PTP1B S-Sulfhydration and Insulin Receptor Substrate 1 Phosphorylation in Insulin- and Metformin-Treated Cells

**Figure 3:**
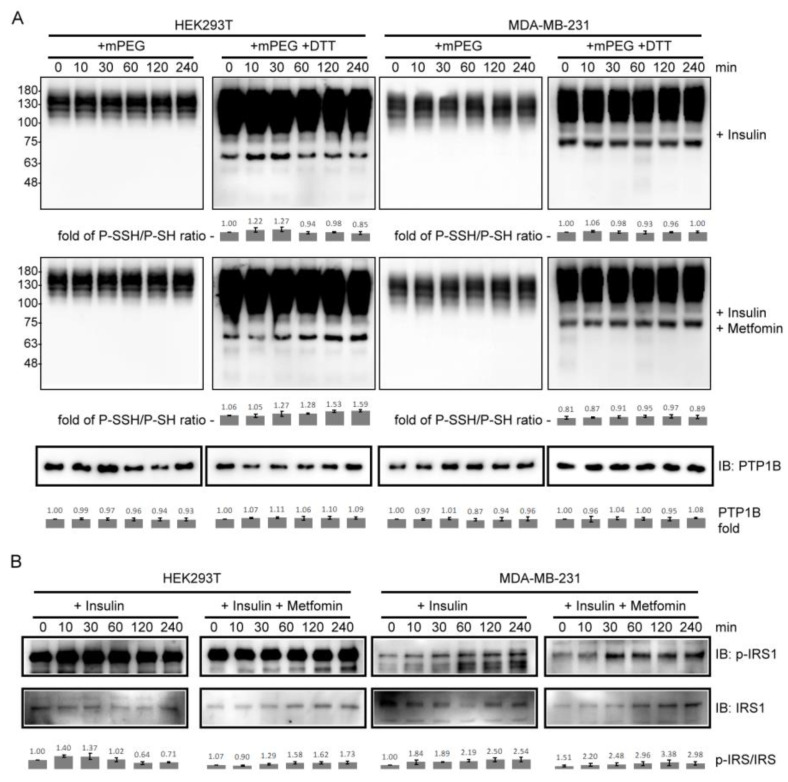
The levels of PTP1B S-sulfhydration and IRS1 phosphorylation in insulin- or combined insulin and metformin-treated HEK293T and MDA-MB-231 cells. (**A**) The immunoblots of PTP1B for detecting S-sulfhydrated PTP1B. (**B**) Immunoblots of phospho-IRS1 (p-IRS1) and IRS1. Insulin: 10 ng/mL insulin; metformin: 10 mM metformin (15 mM metformin for MDA-MB-231 cells). P-SSH/P-SH ratio was derived from dividing the value from lower band in the blot (+mPEG +DTT) by the value from corresponding band in the blot (+mPEG). p-IRS-1/IRS1 ratio was derived by dividing the value from band in the p-IRS1 blot by the value from corresponding band in the IRS1 blot. The fold change was derived by normalizing the value from each sample to that from the sample at 0 min (untreated control cells) (*n* = 3).

To determine whether PTP1B S-sulfhydration is involved in insulin signaling with metformin and examine the difference between cell types, we applied PEG-switch method to detect the S-sulfhydrated PTP1B in insulin- and metformin-treated HEK293T and MDA-MB-231 cells. As a result, the levels of S-sulfhydrated PTP1B reached their maximum at 30 min and decreased from 60 to 240 min in insulin-treated HEK293T cells, but showed no difference in the insulin-treated MDA-MB-231 cells over time (Figure 3A). Interestingly, the co-treatment with insulin and metformin changed the trend of S-sulfhydrated PTP1B in HEK293T cells but only had a slight effect on the trend for the MDA-MB-231 cells (Figure 3A). To further investigate the effect of PTP1B S-sulfhydration, the Insulin receptor substrate 1 (IRS1) phosphorylation was also monitored in these samples. The levels of phospho-IRS1 reached their maximum in the time interval between 10 and 60 min, and decreased from 60 to 240 min in insulin-treated HEK293T cells. However, the levels of phospho-IRS1 reached their maximum at 240 min in insulin- and metformin-co-treated HEK293T cells (Figure 3B). In MDA-MB-231 cells, the trend of IRS1 phosphorylation with insulin treatment was similar to that with co-treatment with insulin and metformin (Figure 3B). The results showed that PTP1B S-sulfhydration was correlated with phospho-IRS1 during insulin stimulation with and without the presence of metformin in HEK293T cells, but not MDA-MB-231 cells, and metformin only specifically affected the change in PTP1B S-sulfhydration and IRS1 phosphorylation in HEK293T cells.

### 2.4. Increased ROS in Insulin-Treated Cells

**Figure 4:**
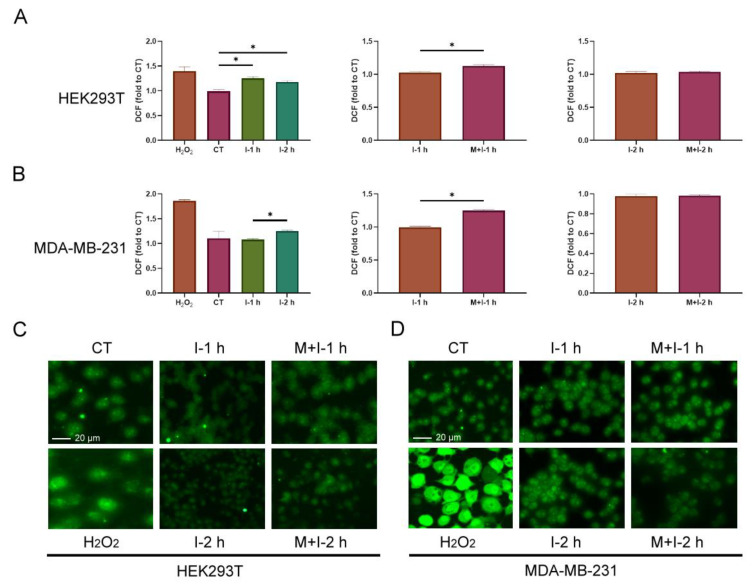
The change in cellular ROS in insulin or combined insulin and metformin-treated HEK293T and MDA-MB-231 cells. The cellular ROS in (**A**) HEK293T and (**B**) MDA-MB-231 cells with each indicated treatment determined by flow cytometry. The fluorescence microscopy image of cellular ROS in (**C**) HEK293T and (**D**) MDA-MB-231 cells with each indicated treatment. CT: control; I: 10 ng/mL insulin; M + I: 10 mM metformin (15 mM metformin for MDA-MB-231 cells) + 10 ng/mL insulin; H_2_O_2_: 0.5 mM H_2_O_2_. The results with significant difference at *p* < 0.05 are indicated by * (*n* = 3). The fold change in DCF was derived from dividing the fluorescence intensity from each sample by that from one of CT sample.

The formation of protein persulfide is a process of oxidation, and the cellular redox state may be involved in the regulation of S-sulfhydrated PTP1B under insulin stimulation. Therefore, we firstly examined ROS formation in both cell lines with treatment with insulin and metformin. The treated cells were stained with DCFH-DA and analyzed by flow cytometry and fluorescence microscopy. According to the results, insulin induced an increase in ROS compared to the control when HEK293T cells were treated with insulin for 1 and 2 h, and the co-treatment with insulin and metformin for 1 h enhanced the level of ROS compared to the treatment with insulin for 1 h (Figure 4A,C). Similarly, insulin only induced a slight increase in ROS compared to the control when MDA-MB-231 cells were treated with insulin for 2 h, but the co-treatment with insulin and metformin for 1 h still enhanced the level of ROS compared to the treatment with insulin for 1 h (Figure 4B,D). Thus, insulin provoked the cellular ROS in both cell lines in slightly different extents, and metformin certainly enhance the increase in both cellular ROS during insulin stimulation.

### 2.5. The Regulation of Cellular H_2_S Using Insulin and Metformin in Both Cell Lines

**Figure 5:**
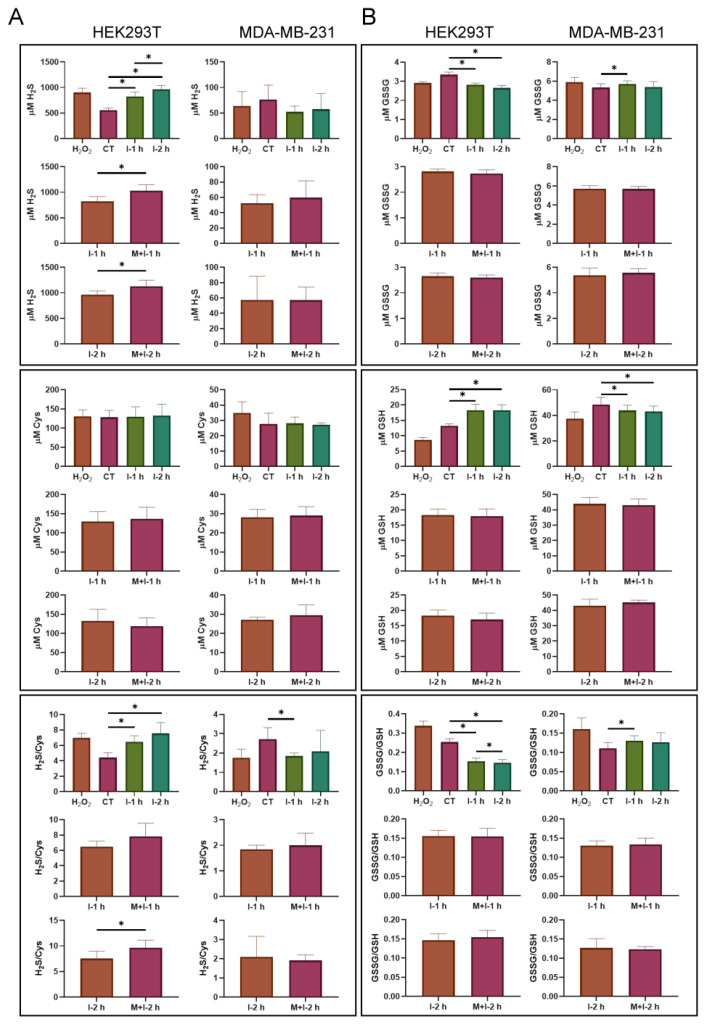
The change in cellular H_2_S, cysteine, GSSG and GSH in insulin or combined insulin and metformin-treated HEK293T and MDA-MB-231 cells. (**A**) The levels and ratio of cellular H_2_S and cysteine. The concentrations (μM in cells) were determined by the detected derivatives of H_2_S and cysteine with LC-MS. (**B**) The levels and ratio of cellular GSSG and GSH. The concentrations (μM in cells) were determined by the detected GSSG and derivatives of GSH with LC-MS. H_2_O_2_: 0.5 mM H_2_O_2_; CT: control; I: 10 ng/mL insulin; M + I: 10 mM metformin (15 mM metformin for MDA-MB-231 cells) + 10 ng/mL insulin. The results with significant difference at *p* < 0.05 are indicated by *. (*n* = 6).

To further understand the formation of PTP1B persulfides, we performed LC-MS analysis to measure the content of thiol metabolites in these cell models. The samples were prepared from insulin- and metformin-treated cells by extracting the metabolites and carrying out chemical derivatization with ^13^C_6–_2-IAN. The levels of H_2_S and cysteine in the cells were measured by detecting the derivatives of H_2_S with *m*/*z* 335.1221 [+Na] and cysteine with *m*/*z* 261.0994 [+H] (Figure S1). As a result, the levels of H_2_S in the HEK293T cells were 19.6-fold those in the MDA-MB-231 cells, on average, and were significantly affected by insulin, while metformin further enhanced the generation of H_2_S along with insulin stimulation at 1 and 2 h. Moreover, the levels of cysteine in HEK293T cells were 5.4-fold those in MDA-MB-231 cells, on average, and the levels of cysteine in both cell lines were not affected by insulin or metformin. The ratios of H_2_S/cysteine in HEK293T cells were also higher than those in MDA-MB-231 cells under overall conditions (Figure 5A). These results indicate that HEK293T cells have a high capacity of H_2_S and cysteine, which is susceptibly modulated by insulin and metformin, compared to MDA-MB-231 cells.

### 2.6. The Regulation of Cellular Glutathione by Insulin and Metformin in Both Cell Lines

The oxidized glutathione (GSSG), with m/z 613.1594 [+H], and GSH-^13^C_6_-2-IAN derivatives, with *m*/*z* 447.1634, were also measured during LC-MS analysis. The levels of GSSG and reduced glutathione (GSH) in MDA-MB-231 cells were generally higher than those in HEK293T cells, and were barely affected by insulin and metformin. However, treatment with insulin or co-treatment with insulin and metformin resulted in a significant decrease in GSSG and an increase in GSH in the HEK293T cells. The ratios of GSSG/GSH remained similar in the untreated cells for each cell line, but decreased significantly in insulin-treated or insulin and metformin-co-treated HEK293T cells. However, metformin had no effect on the levels of GSSG and GSH during insulin stimulation (Figure 5B). Therefore, MDA-MB-231 cells contained a higher capacity of GSSG and GSH than HEK293T cells, which could buff the formation of H_2_S and protein persulfides.

### 2.7. The Change in PTP1B Activity in Insulin- and Metformin-Treated Cells

**Figure 6:**
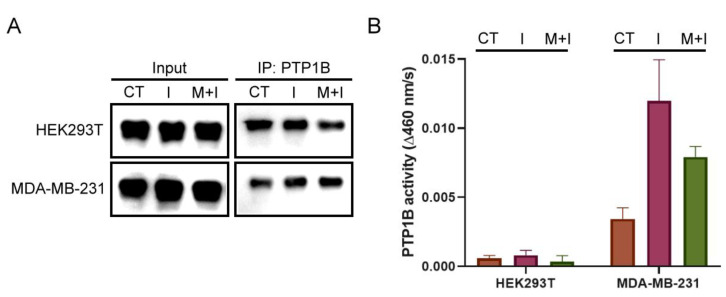
In situ PTP1B activity in insulin- or combined insulin and metformin-treated HEK293T and MDA-MB-231 cells: (**A**) Immunoprecipitation of PTP1B from the insulin- or combined insulin and metformin-treated HEK293T and MDA-MB-231 cell lysate. (**B**) The PTP1B activity of each sample of immunoprecipitation. I: 10 ng/mL insulin for 2 h; M + I: 10 mM metformin (15 mM metformin for MDA-MB-231 cells) + 10 ng/mL insulin for 2 h. (*n* = 3).

Although we found the states of PTP1B S-sulfhydration were related to the levels of IRS1 phosphorylation, the effects on PTP1B activity in those treated cells remained unknown. Therefore, the PTP1B were isolated from each lysate of insulin- and metformin-treated cells by immunoprecipitation and then subjected to immunoblotting and PTP activity assay. The immunoblots of PTP1B were used to determine the content of each pool of immunoprecipitated PTP1B (Figure 6A). The results of PTP activity assay showed that the PTP1B activity increased when cells were treated with insulin for 2 h in both cell lines compared to the control cells. In addition, co-treatment with insulin and metformin downregulated the PTP1B activity compared to treatment with insulin in both cell lines. The PTP1B activity in MDA-MB-231 cells was much higher overall than that in HEK293T cells under all conditions (Figure 6B). These results indicate that metformin treatment can result in the suppression of PTP1B activity, and other regulations are still involved in activating PTP1B activity during insulin stimulation. Moreover, the lower overall PTP1B activity in HEK293T cells could result from the higher levels of H_2_S and S-sulfhydrated PTP1B compared to MDA-MB-231 cells.

## 3. Discussion

Among the molecular mechanisms of H_2_S influencing cellular functions, the occurrence of protein S-sulfhydration is involved in regulating protein functions. More and more cases of protein S-sulfhydration and the functioning mechanism have been described. Recently, it was found that dynamin-related protein 1 (Drp1) can be S-sulfhydrated by CSE to ameliorate heart dysfunction. The study provides a novel mechanism whereby H_2_S S-sulfhydration of Drp1 at Cys607 is able to prevent heart failure through modulating its activity and mitochondrial translocation [31]. S-sulfhydration of methylenetetrahydrofolate reductase (MTHFR) has been described as increasing the enzyme activity and restricting the progress of hyperhomocysteinemia [32]. S-sulfhydration on the active site of glycolytic enzyme glyceraldehyde-3-phosphate dehydrogenase (GAPDH) leads to its redistribution into the nucleus, and the nuclear localization of GAPDH is critical for H_2_S-mediated activation of autophagy by disrupting CCAR2-SIRT1 interaction [16]. Therefore, S-sulfhydration does indeed contribute a crucial effect for conducting H_2_S-mediated functions, and the development of a useful method for screening S-sulfhydrated proteins could be helpful to accelerate the identification of important H_2_S-mediated downstream proteins. In this article, we proposed a feasible and convenient method for detecting protein S-sulfhydration, and found a linkage between insulin signaling and PTP1B modification via cellular redox states. The PEG-switch method was originally proposed for detecting protein oxidation in a previous study, and we further modified the method to specifically detect protein S-sulfhydration. With our method, the evaluation of protein S-sulfhydration under specific stimulation becomes feasible. When comparing the PTP1B S-sulfhydration and related redox mediators in two cell lines, the results indicated that the level of ROS, H_2_S, and GSH is relevant to the extent of PTP1B S-sulfhydration under insulin signaling. The increase in ROS and H_2_S could promote the formation of protein persulfide in this progression. We found that insulin can induce an increase in ROS, H_2_S, and GSH, and meanwhile, metformin can boost the production of H_2_S in the early stage under insulin stimulation in HEK293T cells. However, insulin and metformin showed moderate and reversed effects on the change in H_2_S, and GSH in MDA-MB-231 cells. The different response between these two cell lines implies that insulin may result in a spectrum of PTP1B S-sulfhydration, depending on the homeostasis of thiol compounds in regulating the level of H_2_S. Therefore, our findings could further help to understand the role and regulation of H_2_S in insulin signaling in different cell types and tissues.

According to our findings, PTP1B S-sulfhydration in samples was able to be determined by applying the PEG-switch method (Figure 1 and Figure 2), the concept of which is similar to the method developed by Sen et al. (2012) [17]. The PEG-switch method is relatively simple compared to methods that utilize the biotin switch strategy, and is theoretically feasible for identifying the S-sulfhydration of any proteins of interest. Although this method is theoretically practical for the detection of the S-sulfhydration of any proteins, certain circumstances still need to be considered, such as the molecular weight, numbers of cysteine residues, and abundance of target protein. Proteins with high molecular weight and low cysteine residue numbers may result in an insignificant gel retardation. However, proteins containing a large number of cysteine residues may cause a smeared stain or a loss of signal, especially for low-abundance proteins. Moreover, the labeled PEG could resist antibody binding, causing a loss of signal. Except for these limitations, the method is still practical for providing a simple and quick solution for detecting protein S-sulfhydration.

PTP1B is known to be susceptible to being S-sulfhydrated at Cys215-SH to form Cys215-SSH in response to ER stress, maintaining PERK in its active and phosphorylated form [12]. We found that recombinant PTP1B was susceptible to being S-sulfhydrated during in vitro incubation with Na_2_S (Figure 2A). PTP1B activity was inhibited by the in vitro reaction, and could be recovered by applying a reducing agent, such as DTT (Figure 2B). These results suggests that the activity of PTP1B can be reversibly regulated in the form of S-sulfhydration, which may be related to the various cytoprotective functions of H_2_S [6]. Theoretically, replenishment of Na_2_S is incapable of generating a persulfide on a Cys-SH residue of protein [33]. Oxygen dissolved in the mixture could be a trigger for catalyzing the formation of PTP1B-SSH in the in vitro reaction. Therefore, under strict anaerobic conditions, the addition of Na_2_S should not cause the formation of persulfide. In insulin-treated cells, the levels of S-sulfhydrated PTP1B varied with the progression of time, and the trend changed apparently with the presence of metformin in HEK293T cells (Figure 3A). A similar trend of IRS1 phosphorylation to PTP1B S-sulfhydration was observed in these insulin- and metformin-treated cells (Figure 3B). These results imply that one mechanism for controlling the level of phospho-IRS1 is mediated by the level of PTP1B S-sulfhydration. The increase in S-sulfhydrated PTP1B decreases the total activity of PTP1B in cells during insulin stimulation. Interestingly, metformin caused a differential efficacy in modulating PTP1B S-sulfhydration and IRS1 phosphorylation between HEK293T and MDA-MB-231 cells. The variation in IRS1 phosphorylation should be partially controlled by the regulation of PTP1B S-sulfhydration, which is one potential mechanism for regulating the downstream protein tyrosine phosphorylation during insulin action. The results imply that the differences in response to insulin stimulation and drug action may come from the specific signaling configuration and the influence of PTP1B S-sulfhydration in each cell type.

Protein S-sulfhydration is considered to be a chemical modification of low-pKa sulfhydryl groups by ROS and H_2_S [9,10,11,12]. Based on our data, it can be seen that the levels of ROS generally increased in insulin-treated cells for both cell lines, and metformin enhanced the increase in ROS around that time point with 1 h insulin treatment (Figure 4). However, the levels of H_2_S responded significantly to treatment with insulin alone and co-treatment with metformin and insulin in HEK293T cells, but not in MDA-MB-231 cells (Figure 5A). The increase in ROS and H_2_S in cells creates a chemical environment that promotes the level of S-sulfhydrated PTP1B. In addition, the induction to increase the content of H_2_S in HEK293T cells may further explain the difference in PTP1B S-sulfhydration in HEK293T and MDA-MB-231 cells. Compared to the level of H_2_S and cysteine in MDA-MB-231 cells, on average, the content in HEK293T cells is definitely higher. Cysteine, as the pivot precursor, can be catalyzed by CBS and CSE to generate H_2_S alone or accompanied by homocysteine [1,2,5], and high cysteine capacity surely enables cells to freely produce H_2_S. Therefore, the levels of H_2_S in HEK293T cells were replenished during insulin stimulation with or without the presence of metformin, but the levels of H_2_S in MDA-MB-231 cells slightly decreased, instead (Figure 5A). The slight decrease in H_2_S and cysteine in MDA-MB-231 cells also implies that H_2_S and cysteine are consumed and insufficiently replenished during the process under insulin action. The extremely high concentration of H_2_S determined in the HEK293T is unusual (Figure 5A). These results may be true, but may also arise due to the procedure of metabolite sample preparation. In our previous study, we found that the order of derivatizing thiol metabolites with 2-IAN from HeLa cells resulted in differing levels of cysteine [34]. Therefore, we were unaware that the difference in the quantification of thiol metabolites resulted from the order of derivatization when processing HEK293T cells. Although the procedure may result in deviations during quantification, by performing sample preparation in a consistent manner, the changes induced by insulin stimulation should still be comparable.

The enhanced increase in ROS by co-treating cells with metformin described by our results contradicts the results reported in previous studies. Batandier, C. et al. found that ROS production induced by a reverse-electron flux at respiratory-chain complex 1 can be hampered by metformin [35,36]. In addition, Beth Kelly et al. found that metformin can inhibit the production of ROS from NADH:Ubiquinone oxidoreductase in lipopolysaccharide (LPS)-activated macrophages [35,37]. Notably, in these studies, the results were obtained from the samples with long-term metformin treatment, e.g., 24 h. Using co-treatment with insulin and metformin in our study was to mimic the condition of taking metformin after meal and to observe the effects of metformin within 4 h. The increase in ROS is basically due to the action of insulin, and metformin boosted the increase in ROS at 1 h and reached the same level at 2 h as observed with the insulin treatment (Figure 4). Batandier, C. et al. also reported that rotenone can induce an increase or a decrease in mitochondrial ROS production, depending on whether glutamate–malate or succinate are available as respiratory-chain substrates, respectively [35,36]. Similarly, the presence of insulin and metformin could induce a transient oxidative stress, depending on the available substrates, such as the increase in the number of metabolite intermediates of glycolysis and tricarboxylic acid cycle due to the increasing glucose uptake. Therefore, the effect of metformin on ROS regulation should be dynamic and at least related to the cellular metabolism.

**Figure 7:**
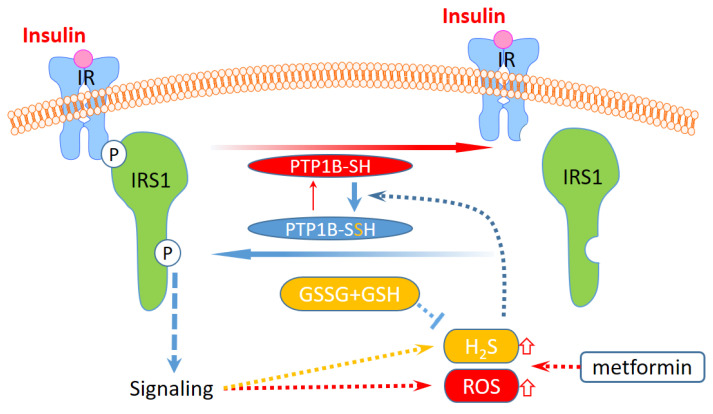
A putative model of differential regulation for insulin signaling in different types of cells via ROS/H_2_S mediated PTP1B S-sulfhydration. The level of H_2_S and ROS could increase in response to insulin signaling, and the increases could be enhanced by metformin and may be suppressed under a high level of glutathione. The increases in H_2_S and ROS can further induce PTP1B S-sulfhydration to suppress this activity by removing the phosphorylation from IRS1. IR: insulin receptor; IRS1: insulin receptor substrate 1.

Notably, the levels of GSH and GSSG in MDA-MB-231 cells are higher than those in HEK293T cells, on average, and are less affected by treatment with insulin or metformin (Figure 5B). Conversely, a decrease in GSSG and an increase in GSH in response to insulin were observed in HEK293T cells. The GSSG/GSH ratio in HEK293T cells also decreased with the insulin treatment, but that in MDA-MB-231 cells increased only slightly. The results suggest that the redox state and the content of glutathione are different in both cell lines, and may result in a differentially buffered pool to quench ROS and H_2_S. Therefore, the high capacity of GSSG and GSH in MDA-MB-231 cells is able to stably maintain the levels of ROS and H_2_S to minimize the change in PTP1B S-sulfhydration. In Figure 6, the overall PTP1B activity in HEK293T cells was apparently lower than that in MDA-MB-231 cells. These results further confirm the relation of H_2_S level and protein S-sulfhydration. Thus, the composition of cellular ROS, H_2_S, cysteine, and glutathione is relevant to the protein S-sulfhydration. Cells activated by insulin will control PTP1B S-sulfhydration mediated by this system to regulate PTP1B activity and downstream protein tyrosine phosphorylation. The regulation can be differentially affected by metformin, depending on cell types due to the difference in metabolism of H_2_S and thiol metabolites (Figure 7).

The sensitizing vs. desensitizing effects of H_2_S on insulin-sensitive tissues is still controversial. H_2_S seems to elicit differential signaling transduction systems in different tissues [38]. For instance, chronic NaHS treatment can promote glucose uptake in both myotubes and adipocytes by increasing insulin sensitivity and ameliorates kidney lesions in type 2 diabetes in diabetic rats [7]. However, NaHS treatment or CSE overexpression impairs glucose utilization and increases gluconeogenesis in hepatocytes [39]. CSE knockout exacerbates obesity and related insulin resistance in high-fat-diet-fed mice and subsequently promotes hepatic gluconeogenesis, which can be reversed by NaHS supplementation [40]. These studies indicate the different effects of H_2_S on signal transduction in different types of tissue or under different physiological conditions. Our findings may be used to further explain the difference in cell types in response to insulin signaling by considering the pool of H_2_S and thiol metabolites. Therefore, cellular signaling could be differentially regulated by S-sulfhydration of PTP1B or other proteins, depending on the homeostasis of the redox state and the H_2_S pool in different tissues.

## 4. Materials and Methods

### 4.1. Cell Culture

A human embryonic kidney 293T (HEK293T) cell line and human breast cancer cell line (MDA-MB-231) were purchased from American Type Culture Collection (ATCC, Cambridge, MA, USA). Cells were cultured in Dulbecco’s modified Eagle’s medium (DMEM), a high-glucose medium containing 10% fetal bovine serum (FBS) in a 5% CO_2_ atmosphere at 37 °C. Cells treated with 10 ng/mL insulin (Calbiochem, San Diego, CA, USA), metformin (10 mM for HEK293T cells; 15 mM for MDA-MB-231) (Sigma-Aldrich, St. Louis, MO, USA), or 0.5 mM H_2_O_2_ were sampled for further analysis.

### 4.2. Sample Preparation for Detecting S-Sulfhydrated Proteins

Each well of 1 × 10^6^ cells (6-well plate) was combined with 100 μL of 2xTTE buffer (40 mM Tris-HCl, pH 8.0, 10 mM EDTA, and 2% Triton X-100) and collected into a microtube with further disruption by transient sonication. The cell lysate was obtained by collecting the supernatant from lysed cells with centrifugation at 14,000× *g* for 10 min. Thirty microliters of cell lysate was mixed with 30 μL of ultrapure water or 1 mM mPEG (methoxypolyethylene glycol maleimide ≥90% (NMR), 5000 Da, 63187, Sigma-Aldrich) water solution and incubated at 25 °C for 1 h. The introduction of mPEG to cell lysate should be done as soon as possible; otherwise, directly lysing cells with mPEG-containing lysis buffer is recommended. After incubation, twenty microliters of mixture was added to 20 μL of 2XSDS and 2XSDS/DTT (containing 200 mM dithiothreitol) sample buffer, respectively, and then heated in a water bath at 100 °C for 5 min. The sample was then subjected to SDS-PAGE and immunoblotting to determine the S-sulfhydrated proteins using the PEG-switch method (Figure 1). FaDu cells were treated with 5 mM diamide (Sigma-Aldrich) for 5 min, and then the nucleolin S-sulfhydration was analyzed using the PEG-switch method with anti-C23 (nucleolin) antibody (Santa Cruz).

### 4.3. In Vitro PTP1B S-Sulfhydration

Recombinant human PTP1B protein (Active) ab51277 (abcam, Cambridge, UK) was incubated in solution containing 5 mM Tris-HCl, pH 7.5, 0.4 mM beta mercaptoethanol, 0.2 mM DTT, and 0.2 mM EDTA with 0.1 μg/μL protein in 0, 0.25, and 0.5 mM Na_2_S (Sodium sulfide nonahydrate; Alfa Aesar, Ward Hill, MA, USA), respectively, at 25 °C for 10 min. Four microliters of mixture were mixed with 4 μL of 5 mM mPEG water solution for 1 h incubation at 25 °C. One microliter of mixture or 4 μL of mPEG-labeled sample was resolved under non-reducing or reducing SDS-PAGE, and the gel was further stained by silver staining. Meanwhile, each mixture was also added to 9 volumes of ultrapure water or 9 volumes of 10 mM DTT, and each 10 uL of diluent was subjected in turn to PTP activity assay (BioVision, Milpitas, CA, USA).

Ten microliters of HEK293T cell lysate (2 mg/mL of total proteins in 2xTTE buffer) were mixed with 10 μL of ultrapure water or 0.5 and 1 mM Na_2_S, respectively, for 30 min incubation at 25 °C. Nine microliters of HEK293T cell lysate (2 mg/mL of total proteins in 2xTTE buffer) were mixed with 1 μL of ultrapure water, 10 mM H_2_O_2_, and 10 mM Tris(2-carboxyethyl)phosphine hydrochloride (TCEP, Sigma-Aldrich), respectively, for 30 min incubation and then added to 10 μL of ultrapure water or 1 mM Na_2_S for 30 min incubation at 25 °C. Then, 10 μL of mixture was combined with 10 μL of pure water or 5 mM mPEG for 1 h incubation at 25 °C. Four microliters of mixture were resolved under non-reducing or reducing SDS-PAGE, and the results were obtained by immunoblotting for PTP1B.

### 4.4. Protein Sample Preparation

Each well of 1 × 10^6^ cells (6-well plate) was combined with 100 μL of RIPA buffer (Apolo Biochemical Inc., Hsinchu, Taiwan) containing phosphatase inhibitor (PhosSTOP™, Sigma-Aldrich) and collected in a microtube with further disruption by transient sonication. The cell lysate was obtained by collecting the supernatant from lysed cells with centrifugation at 14,000× *g* for 10 min. The sample was then subjected to SDS-PAGE and immunoblotting.

### 4.5. Immunoblotting

Proteins in the resolved SDS-PAGE gel were transferred onto a PVDF membrane by electrophoretic transfer. The PVDF membrane was blocked with 3% skim milk in PBS for 1 h, and then the target proteins were probed using the specific antibody, anti-PTP1B (D-4, Santa Cruz biotechnology, Dallas, TX, USA), anti-IRS1 (E-12, Santa Cruz biotechnology, Dallas, TX, USA), or anti-IRS1 (phospho Y632, abcam, England and Wales, UK) antibody in 1% BSA overnight at 4 °C. After washing three times with PBS, for 10 min each time, the membrane was further incubated in horseradish peroxidase-conjugated second antibody (PerkinElmer, Waltham, MA, USA) for 1 h. The membrane was washed three times with PBS for 10 min each time, and the signals were detected using the standard ECL protocol (PerkinElmer, Waltham, MA, USA).

### 4.6. Flow Cytometry

For flow cytometry, HEK293T and MDA-MB-231 cells (1 × 10^6^ cells/well) were seeded on a 6-well plate and cultured overnight. Each well of cells treated with H_2_O_2_, insulin, or insulin plus metformin was washed with 2 mL of PBS twice, trypsinized, and suspended in 0.5 mL PBS containing 1 μM DCFH-DA (2′,7′-dichlorofluorescein diacetate, Sigma-Aldrich) for the flow cytometry analysis with a BD FACSCanto flow cytometer (BD Bioscience). The geometric mean of DCF fluorescent intensity was used to determine the intracellular ROS levels.

### 4.7. Fluorescence Microscopy

For fluorescence microscopy, HEK293T and MDA-MB-231 cells (1 × 10^4^ cells/well) were seeded on a 24-well plate and cultured overnight. Each well of cells treated with H_2_O_2_, insulin, or insulin plus metformin was washed twice with 2 mL of PBS and incubated with 0.5 mL of 1 μM DCFH-DA (Sigma-Aldrich) in PBS at 37 °C for 30 min in a dark environment. Then, the stained cells were washed twice with 2 mL of PBS and a fluorescence microscope (magnification ×20 and ×40; Zeiss Corporation) was applied to capture the images. Fluorescence was detected by setting excitation at 488 nm and emission at 610 nm, and the microscope was equipped with an FITC filter. The images were processed using Zeiss Axio Vision software.

### 4.8. Metabolite Sample Preparation

Each well of 1 × 10^6^ cells in a 6-well plate was combined with 100 μL of ultrapure water and disrupted by transient sonication. After centrifugation at 14,000× *g* for 10 min, 100 μL of supernatant was collected in a new microtube with the addition of 400 μL of 100% methanol and kept at −80 °C for 2 h. The mixture was warmed to 25 °C and then centrifuged at 14,000× *g* for 10 min. The supernatant was divided into aliquots of 200 μL and dried in a vacuum concentrator.

### 4.9. Metabolite Sample Derivatization

The chemical derivatization of the extracted samples and metabolite standards was performed following a procedure described previously [34]. In brief, the dry sample was combined with 30 μL of 0.2 mM ^13^C_6_-2-Iodoacetanilide (^13^C_6_-2-IAN; Cambridge Isotope Laboratories) in 20 mM NaHCO_3_/Na_2_CO_3_, pH 9.4, buffer and incubated at 60 °C for 2 h. The reactant was then added to 30 μL of 2% formic acid. The derivatized sample was subjected to centrifugation at 14,000× *g* for 10 min, and the supernatant was transferred to an insert vial and kept at 10 °C queuing for LC-MS analysis.

### 4.10. LC-ESI-MS Analysis

An ultra-performance liquid chromatography (UPLC) system (ACQUITY UPLC I-Class, Waters) and an ESI/APCI source of 4 kDa quadrupole time-of-flight (TOF) mass spectrometer (Waters VION, Waters, Milford, MA, USA) were used to perform LC-ESI-MS analysis. Separation was conducted with reversed-phase liquid chromatography (RPLC) on a BEH C18 column (2.1 × 100 mm, Waters) with the flow rate of 0.2 mL/min at a column temperature of 35 °C and a 7.5 μL sample injection. The elution started from 99% mobile phase A (ultrapure water + 0.1% formic acid) and 99% mobile phase B (100% methanol + 0.1% formic acid), held at 1% B for 0.5 min, raised to 95% B in 5.5 min, held at 95% B for 1 min, and then lowered to 1% B in 1 min. The column was equilibrated by pumping 1% B for 4 min. LC-ESI-MS chromatogram were acquired by ESI+ mode under following conditions: capillary voltage of 2.5 kV, source temperature of 100 °C, desolvation temperature at 250 °C, cone gas maintained at 10 L/h, desolvation gas maintained at 600 L/h, and acquisition by MS^E^ mode with the range of *m*/*z* 100–1000 and 0.5 s scan time. The acquired data were processed by UNIFI software (Waters), and the concentration of metabolite was calculated from the integrated peak area by applying the standard calibration curve.

### 4.11. Immunoprecipitation

Each well of 1 × 10^6^ cells (6-well plate) was combined with 100 μL of RIPA buffer (Apolo Biochemical Inc.) and collected in a microtube with further disruption by transient sonication. The cell lysate was obtained by collecting the supernatant from lysed cells with centrifugation at 14,000× *g* for 10 min. Ninety microliters of lysate was added to 10 μL of anti-PTP1B antibody for 1 h incubation at 25 °C and 20 μL of suspended Pierce protein A agarose was added for a further 1 h of incubation. The agarose was washed with 500 μL of PBS buffer three times and then suspended in 200 μL of PBS for the immunoblotting and PTP activity assay.

### 4.12. PTP1B Activity Assay

The PTP1B activity was measured by using the fluorometric Protein tyrosine phosphatase activity assay kit (BioVision). The recombinant PTP1B and immunoprecipitated PTP1B were applied to the working solution by following the instruction and acquiring the fluorescence from (Ex/Em = 368/460 nm) with the Synergy HT multi-detection microplate reader (Bio-TeK, Winooski, VT, USA). The data were collected with kinetic mode with a 20 s period for 10 min for calculate the change in fluorescence with time.

### 4.13. Statistics

All experiments were performed at least three times. The protein levels were determined by quantifying each signal of immunoblots using ImageJ (National Institutes of Health). Statistical comparisons were analyzed by one-way ANOVA with multicomparison tests and unpaired t-test using GraphPad Prism software version 8.0.1 (GraphPad Software, San Diego, CA, USA). Significance was considered at probability error (*p*) <0.05, and all *p* values were two-tailed. The plot was created using the mean and standard deviation for error bars.

## 5. Conclusions

Since H_2_S was classified as a gaseous signaling molecule, the mechanisms behind miscellaneous functions have still remained to be revealed. One of the main aspects is to study the S-sulfhydration of proteins. In this case, we applied the PEG-switch method to determine the levels of PTP1B S-sulfhydration, and confirmed that PTP1B S-sulfhydration mediated by H_2_S was involved in the regulation of insulin signaling. The contents of ROS, H_2_S, cysteine, GSSG, and GSH were relevant to the process of PTP1B S-sulfhydration. These changes were found to be regulated by insulin stimulation and differentially affected by metformin, depending on cell type. Therefore, the PTP1B activity could be regulated by cellular H_2_S and redox state in response to insulin stimulation. Moreover, the function of metformin in reducing insulin resistance could also result from the suppression of PTP1B activity by S-sulfhydration. Basically, the usage of glucose is different in types of cells and tissues. Beyond the configuration of protein phosphorylation signaling cascade, we suggest that a linkage between H_2_S homeostasis and redox regulation for PTP1B S-sulfhydration may play a role in maintaining insulin signaling. Hopefully, our findings could further help understand the role and regulation of H_2_S for insulin functioning in different cell types and tissues.

## Data Availability

Data is contained within the article or Supplementary Material.

## Supplementary Materials

The following supporting information can be downloaded at: https://www.mdpi.com/article/10.3390/ijms24032898/s1.
