# Supplementary material for: H2S- and Redox-State-Mediated PTP1B S-Sulfhydration in Insulin Signaling
Source: Int J Mol Sci. 2023 Feb 2;24(3):2898. doi: 10.3390/ijms24032898 (PMC9917502; doi:10.3390/ijms24032898)
Supplement: Supplementary file 1 [file ijms-24-02898-s001.zip › ijms-2117728-supplementary.pdf]

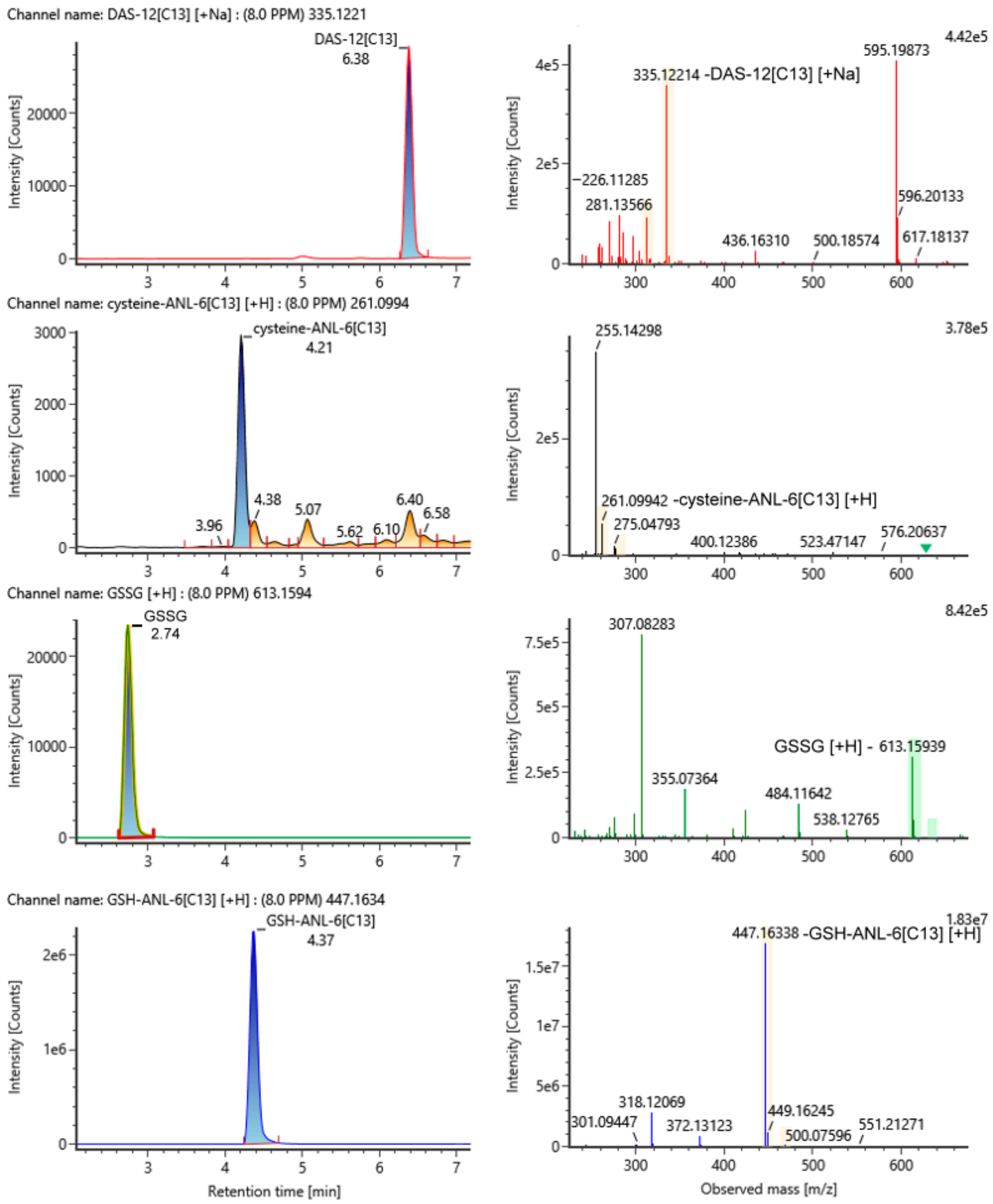

Figure S1. Representative chromatogram and mass spectrum of detected thiol metabolites. DAS-12[C13]: H_2S-$^{13}C_6$-2-IAN derivative; cysteine-ANL-6[C13]: cysteine-$^{13}C_6$-2-IAN derivative; GSSG: oxidized GSH; GSH-ANL-6[C13]: GSH-$^{13}C_6$-2-IAN derivative. [+Na]: compound-sodium adducts; [H+]: compound-proton adducts.
